# COVID-19 pandemic: what factors compromised the mental capacity to work of nursing technicians?

**DOI:** 10.1590/0034-7167-2022-0783

**Published:** 2024-06-28

**Authors:** Katia Maria Rosa Vieira, Francisco Ubaldo Vieira, Zélia Zilda Lourenço de Camargo Bittencourt

**Affiliations:** I Universidade Estadual de Campinas. Campinas, São Paulo, Brazil; II Instituto Federal de São Paulo. Campinas, São Paulo, Brazil

**Keywords:** Pandemics, COVID-19, Working Conditions, Occupational Health, Nursing, Pandemias, COVID-19, Condiciones de Trabajo, Salud Laboral, Enfermería

## Abstract

**Objectives:**

to identify the factors that influenced the mental capacity to work of nursing technicians during the COVID-19 pandemic.

**Methods:**

a cross-sectional study, carried out at two reference hospitals in assisting the population during the COVID-19 pandemic. A total of 237 professionals from Intensive Care Units participated and answered a questionnaire. Multiple linear regression models were used to assess the correlation between mental capacity to work and variables related to risks of contamination, institutional support and health.

**Results:**

lack of COVID-19 tests, lack of knowledge of routine, absences in 2021 and mental illness contributed to worse mental capacity to work. Management embracement and physical capacity were considered protective factors.

**Conclusions:**

reduced work capacity in relation to mental demands can affect professional performance and quality of care, with implications for patients and health institutions.

## RESUMEN

**Objetivos:**

identificar los factores que influyeron en la capacidad mental para el trabajo de técnicos de enfermería en el contexto de la pandemia de COVID-19.

**Métodos:**

estudio transversal, realizado en dos hospitales de referencia que atienden a la población durante la pandemia de COVID-19. Participaron 237 profesionales de Unidades de Cuidados Intensivos que respondieron un cuestionario. Se utilizaron modelos de regresión lineal múltiple para evaluar la correlación entre la capacidad mental para el trabajo y variables relacionadas con riesgo de contaminación, apoyo institucional y salud.

**Resultados:**

falta de pruebas de COVID-19, desconocimiento de la rutina, ausencias en 2021 y enfermedad mental contribuyeron al empeoramiento de la capacidad mental para el trabajo. Se consideraron factores protectores la recepción por parte de la dirección y la capacidad física.

**Conclusiones:**

una capacidad reducida para trabajar en relación con las demandas mentales puede afectar el desempeño profesional y la calidad de la atención, con implicaciones para pacientes y instituciones de salud.

## INTRODUCTION

Global health was threatened by a disease called Coronavirus Disease-2019 (COVID-19), triggered by a new coronavirus (SARS-CoV-2). Due to its expansion to all continents in the world, except Antarctica, the World Health Organization (WHO) declared it a pandemic on March 11, 2020^(1)^.

During the pandemic, international and national agencies expressed their concern about health professionals’ working conditions, accident prevention and safety culture promotion in institutions^(2-3)^.

Nursing technicians are the category that represents the largest nursing workforce in Brazil, with 1,715,191 professionals (59.5%) with active registrations^(4)^, and had no assistance or financial resources to deal with the impact of their illness or that of their family members during this serious health crisis^(4-5)^.

The COVID-19 pandemic revealed weaknesses in the health system, which presented a shortage of qualified professionals, lung ventilators, personal protective equipment (PPE), essential supplies^(6)^, lack of diagnostic tests^(7)^ and failures in the organization of Intensive Care Units (ICU), functioning beyond their capacity^(8)^.

Studies carried out before the pandemic demonstrated that increased workload intensified physicians’, nurses’ and health assistants’ stress and anxiety, with a decrease in their cognitive functions, reasoning and judgment capacity, with impaired attention and coordination^(9-10)^, with greater chances of failures and work accidents^(11)^.

Research carried out in several countries showed that, during the pandemic, health professionals present symptoms of exhaustion, anxiety, stress, depression and illness^(12-15)^, with reduced functional capacity of nurses with mild symptoms and moderate infections due to COVID-19^(16-17)^ and compromised professional performance due to excessive workload^(18)^, enhanced by absenteeism^(19)^.

This overview took on a complex contour, as, in addition to predisposing nursing technicians to the risk of contamination by the coronavirus, it compromised their ability to work^(10,18,20)^. This concept refers to a dynamic balance between stress, working environment conditions, tools and professional exhaustion^(21)^.

The mental capacity to work of nursing technicians is related to their cognitive functions, which, to provide health care to patients, requires reasoning and judgment capacity to solve problems, memorize information, perform calculations and interpret data.

A study carried out in the United States of America^(22)^ showed that nurses who worked with patients with COVID-19 felt helpless, unprepared and overwhelmed, with high levels of exhaustion, physical symptoms and negative emotions.

International literature reports some support strategies for prevention and intervention in nursing professionals’ mental health. A study carried out in China^(23)^ showed that cognitive strategies based on values and adequate social support facilitated work experiences with patients with COVID-19. Research carried out in the Philippines, in 20 hospitals, revealed that organizational and social support had consistent effects in improving nurses’ anxiety^(24)^.

In the current literature, there are few specific studies aimed at nursing technicians during the COVID-19 pandemic. It is imperative to identify factors associated with mental health and capacity to work in the largest professional category of health care. Understanding this relationship allows to expand their understanding of working conditions, providing support to implement actions to promote these workers’ health and quality of life.

## OBJECTIVES

To identify the factors that influenced the mental capacity to work of nursing technicians during the COVID-19 pandemic.

## METHODS

### Ethical aspects

All ethical procedures were followed, in accordance with Resolution 466/2012 of the Brazilian National Research Council. The project was submitted and approved by the Research Ethics Committee. All participants signed the Informed Consent Form (ICF), respecting nursing professionals’ and institutions’ confidentiality and privacy.

### Study design, place and period

This is a cross-sectional, exploratory, quantitative study, guided by STrengthening the Reporting of OBservational studies in Epidemiology (STROBE) guidelines.

The study was carried out in two hospitals, selected for convenience, which are centers of excellence in medical and hospital care and a reference in serving the population during the COVID-19 pandemic, with the following characteristics: institution 1 - general, public, state, large hospital, teaching and research, tertiary and quaternary level, providing exclusive service to Brazilian Health System (SUS - *Sistema Único de Saúde*) users; institution 2 - general, private non-profit hospital, large, teaching and research, tertiary level, serving patients covered by health plans and private individuals.

The two health institutions are located in the city of Campinas, countryside of São Paulo. Data were collected in person by the researcher from March to June 2022.

### Population or sample: inclusion and exclusion criteria

All nursing technicians from health institutions were invited to participate in the study. At the time of collection, the hospitals had 384 nursing technicians in the ICU, 56 of whom were on vacation, leave or on leave. Only eight subjects did not agree to participate: six from the public hospital and two from the private network.

All nursing technicians who worked for at least six months in the institutions’ ICUs, of both sexes, who provided direct assistance to patients with COVID-19 in the morning, afternoon and night work shifts were included. Professionals who were on vacation, leave or leave were excluded.

### Study protocol

To collect data, a checklist-type questionnaire was developed with objective questions. A pre-test was applied to six subjects from one of the participating institutions, with the instrument subsequently readjusted in semantic and syntax aspects.

The questionnaire consisted of four structured chunks of questions.

Chunk 1 - General and sociodemographic data: sex; age; time of institution; working time in ICU; and number of jobs.

Chunk 2 - Risk factors for contamination by COVID-19, with participants being able to list one or more items: prolonged working hours; accelerated work pace; lack of PPE; poor quality of PPE; excess jobs (two or more); absence and/or delay in vaccination of health professionals; lack of suitable resting place; non-use of PPE; work accidents with biological fluid/respiratory secretion from a patient with COVID-19; lack of secure information; deficit/absence of tests for workers suspected of having COVID-19; work overload; insufficient number of nursing technicians; stress; absences from shifts (absenteeism); lack and/or failure to wash hands; unfamiliarity with the ICU work routine; failure in circulation between contaminated and uncontaminated areas; accidental PPE removal/displacement (mask falling, others); lack of in-service training/education; failure to apron donning/doffing; discontinuous use of PPE due to appearance of skin injuries during the pandemic; failure to dispose of hospital waste; lack and/or failure to clean and disinfect surfaces; contamination during handling of PPE (N95/PFF2 masks).

Chunk 3 - Institutional measures to support health professionals, with participants being able to list one or more items: psychologist support; psychiatrist support; management embracement; multidisciplinary team support; labor gymnastics; and integrative practices.

Chunk 4 - Workers’ health: diseases acquired during the pandemic with a medical diagnosis (response pattern: Yes-No, with a description of diagnosis); absences from work during 2020 and 2021 (response pattern: Yes-No); self-perception of mental and physical capacity for current job in relation to mental (solving problems, performing calculations, memorizing information, analyzing data) and physical demands (perform physical effort, handle patients, carry out transport) compared to before the COVID-19 pandemic (response pattern: Much worse = 1, Worse = 2, Same capacity = 3, Improved = 4 and Much improved = 5).

### Analysis of results, and statistics

After collection, data were typed into a Microsoft Excel^®^ spreadsheet (v.2308), and statistical analysis was performed with the aid of the Minitab 19 program. Multiple linear regression was used, with a gradual procedure and automatic stepwise selection to examine the correlation between the dependent variable mental capacity to work and the set of independent variables, included in the following models:

Model 1 - (Risk of contamination): due to the large number of independent variables (25) related to contamination risk factors (chunk 2), Mallows’ Cp coefficient was used to help balance the number of predictors (variables) that best fit mental capacity to work. A Mallows’ Cp value that is close to the number of predictors plus the constant indicates that the model is relatively unbiased in estimating true and unbiased regression coefficients. For this, Cp ≈ K+1 was used, where k - was the number of variables in the model.

Model 2 - (Institutional support): psychiatric support, psychological support, management embracement, multidisciplinary team support, workplace gymnastics and integrative practices to measure the influence of institutional support on mental capacity to work.

Model 3 - (Health): physical capacity to work, absences in 2020, absences in 2021 and mental illness were included.

Collinearity was assessed by variance inflation factor (VIF), with VIF<5 being interpreted as absence of collinearity. The Shapiro-Wilk test, to verify the normality of residuals, the lack of fit test, to assess data adequacy to the model, the Durbin-Watson test, to verify the autocorrelation of residuals, and the adjusted coefficient of determination (_adj_R^2^), to assess model goodness of fit, were used. For all results, p<0.05 was considered statistically significant.

## RESULTS

A total of 237 nursing technicians (179 women, 58 men) participated in the research, 143 from a public institution and 94 from a private institution, with a mean age of 38.4±9.8 years (mean ± standard deviation), time at the institution of 7.8±6.6 years, working time in the ICU of 6.0±5.4 years and a mean of jobs of 1.5±0.55. Of the participants, 64.3% self-perceived a worsening of their current mental capacity to work, and 67.2%, a worsening of their current physical capacity to work, compared to before the COVID-19 pandemic.

The analysis of the best subsets for the variables incorporated in Model 1, related to the risk of contamination, included stress, lack of resting place, absence of COVID-19 tests, unfamiliarity with the routine, PPE contamination and handling and non-use of PPE due to skin injuries. This was the best subset with six variables, and resulted in Mallows’ Cp = 6.98.

Table 1 shows the results of the multiple linear regression of the variables that made up the three models.

**Table 1:** Multiple linear regression of the mental capacity to work of nursing technicians in relation to the variables contained in the models, Campinas, São Paulo, Brazil, 2023

Variable	Mental capacity to work					
	Model 1 Risk of contamination		Model 2 Institutional support		Model 3 Health	
	Coef	VIF	Coef	VIF	Coef	VIF
Stress	-0.245^^*^^	1.10	-0.234^^*^^	1.10	--	--
Lack of resting place	-0.206	1.10	-0.205^^*^^	1.13	--	--
Absence of COVID-19 tests	-0.294^^*^^	1.10	-0.243^^*^^	1.14	-0.277^†^	1.11
Unfamiliarity with the routine	-0.251	1.10	-0.245	1.10	-0.212^^*^^	1.06
PPE contamination and handling	-0.211	1.10	-0.213	1.13	--	--
Non-use of PPE due to skin injuries	-0.371^^*^^	1.10	-0.393^^*^^	1.13	-0.258	1.10
Management embracement			0.277^^*^^	1.10	0.192^^*^^	1.07
Multidisciplinary team support			0.310^^*^^	1.03	0.206	1.07
Physical capacity to work					0.439^†^	1.12
Absences in 2020					-0.170	1.23
Absences in 2021					-0.263^†^	1.17
Mental illness					-0.366^†^	1.07
R^2^	12.48%		16.42%		47.33%	
R^2^_aj_	10.20%		13.49%		46.58%	
Normality (Shapiro-Wilk)	<0.01		<0.01		>0.100	
Lack of adjustment	0.930		0.378		0.686	
Durbin-Watson	> 0.05		> 0.05		> 0.05	

* *p<0.05; †p<0.01; Coef - slope coefficient; VIF - variance inflation factor; PPE - personal protective equipment.*

The results of the multiple linear regression of the variables that made up the risk of contamination (Model 1) showed low explanatory power (_adj_R^2^=10.2%), revealing that stress, lack of testing for COVID-19 and non-use of PPE due to skin injuries were statistically significant and showed a negative correlation with mental capacity to work.

With the inclusion of the variables that made up institutional support (Model 2), the explanatory power of mental capacity to work improved (_adj_R^2^=13.49%), revealing that management embracement and multidisciplinary team support were statistically significant. Risk of contamination, stress, absence of COVID-19 tests and non-use of PPE due to skin injuries maintained significance, and lack of resting place became part of the model. It is noteworthy that variables related to institutional support showed a positive correlation with mental capacity to work.

When adding health-related variables (Model 3), there was a substantial improvement in the explanatory capacity of mental capacity to work (_adj_R^2^ = 46.58%), revealing that physical capacity to work, absences in 2021 and mental illness were statistically significant. It is noteworthy that, with the inclusion of health-related variables, stress, lack of resting place and PPE contamination and handling are no longer part of the model, and non-use of PPE due to skin injuries and multidisciplinary team support no longer showed statistical significance. With the inclusion of health-related variables, unfamiliarity with the routine became part of the final model.

In the final model, there was no indication that multicollinearity biased the results, as none of VIF scores exceeded 1.3. The residual normality test showed normal distribution (p>0.10), and there was no indication of residual autocorrelation (p>0.05) or lack of data adjustment (p>0.686).

The final model was represented by the following variables: absence of COVID-19 tests; unfamiliarity with the routine; management embracement; physical capacity to work; absences in 2021; and mental illness.

It was observed, in the final model, that the variables incorporated into the risk of contamination had negative correlation coefficients, i.e., they negatively compromised the mental capacity to work. The variable that made up institutional support (management embracement) showed a positive correlation with mental capacity. Finally, the variables that made up the health model showed a positive correlation between physical capacity and mental capacity. Absence in 2021 and mental illness were negatively correlated with mental capacity to work.

## DISCUSSION

The pandemic crisis has had a profound impact on the world, due to the rapid spread of the disease and the challenges posed to health institutions. There was a mismatch in the speed of building scientific evidence and implementing effective actions over time, which often resulted in an increase in the chances of nursing team contamination, with impacts on workers’ physical and mental health as well as quality of care^(25)^.

In Brazil, one of the countries most affected by the pandemic, the situation was worsened due to divergences, misalignment of government actions and different views on how to confront the health crisis. In public management, a protocol was adopted for using chloroquine and hydroxychloroquine to treat patients with COVID-19, led by the Brazilian Federal Government. Due to the lack of scientific support to justify the adoption of this treatment, clashes and contradictions occurred between health professionals, scientific societies and public health authority institutions^(26)^.

Despite divergences, COVID-19 spread throughout the country, with high morbidity and mortality rates^(27)^. Health professionals who were on the front line were committed to assisting patients, with few resources and knowledge^(6)^, high risk of occupational exposure^(2)^, in addition to reduced attention capacity^(9)^ and problem-solving capacity at work^(10)^.

The present study analyzed 25 variables related to the risk of contamination. Only the absence of COVID-19 tests and unfamiliarity with the routine were correlated with mental capacity to work.

To face a health crisis of this magnitude, nursing professionals with physical and mental skills were needed to carry out activities in an environment permeated by inadequate working conditions^(15)^. Research has shown that long working hours^(18)^ and accelerated work pace^(20)^ were associated with reduced work capacity. However, in the present study, these results were not observed.

Health professionals experienced strong emotional stress^(12)^, psychological pressures and uncertainties regarding the proportions of the pandemic^(28)^, mainly due to the lack of diagnostic tests for COVID-19^(29)^, fear of risk of contamination and spread of disease to family members, lack of information and the possibility of mental disorders^(30-31)^.

In the present research, the absence of COVID-19 tests was negatively related to mental capacity to work, causing insecurity and fear of spread of coronavirus, and, when associated with stress, contributed to a reduction in their cognitive functions for work.

This result is consistent with the literature. A study developed in Poland^(32)^ showed that the possibility of testing for COVID-19 had a protective influence on nurses’ mental health. Research in Indonesia^(7)^ with nurses showed an association between stress and the lack of diagnostic testing services for COVID.

The initial uncertainty and dearth of knowledge about COVID-19 has caused severe stress and feelings of panic among the general population^(33)^. Lack of knowledge about the virus’ incubation period, its form of transmission, treatment and safety measures caused feelings of fear and anxiety, with negative impacts on mental health due to uncertainty regarding the future^(34)^.

Nursing work is characterized by well-established routines and protocols in health services. During the pandemic, there was a substantial change in these routines, with an extension of working hours^(35)^, lack of information about the new disease^(29)^, health system’s inability to meet demand^(8)^, frequent training^(13)^, new biosafety protocols^(12)^ and lack of knowledge to care for patients^(36)^.

In the present study, unfamiliarity with the work routine was a variable negatively related to mental capacity to work. During the pandemic, the country was experiencing an infodemic^(37)^ related to prevention and control protocols to combat the virus, which may have increased nursing technicians’ mental illness.

This finding was consistent with a study carried out in China^(38)^, in which nurses who were on the front line of the fight against coronavirus presented fear, anxiety, anger and sadness due to close contact with patients, lack of familiarity with procedures and unusual workplaces. These conditions triggered psychological distress, causing depressive, anxiety and stress symptoms^(14)^ that affected performance and mental capacity to work.

In the present work, among the variables that made up the model related to institutional support, only management embracement showed a positive correlation with mental capacity to work.

The well-being of members of multidisciplinary teams is a shared responsibility between the health institution and the professionals who make up it. In the present study, members of multidisciplinary teams were exposed to numerous stressors in the work environment^(12,15)^, with differences in conduct and difficulties in communication. This made it impossible for multidisciplinary team support to become a protective factor for mental capacity to work.

This result is consistent with a study carried out in Australia^(39)^ during the COVID-19 pandemic with a multidisciplinary team made up of physicians, nurses and support professionals. The findings highlighted that nursing professionals had lower satisfaction with the work environment, and the lack of team support contributed to higher levels of stress, anxiety and exhaustion.

Organizational support has been identified in the literature as a protective factor against adversity and stress among nurses, allowing them to maintain their well-being and mental health^(24)^.

In this research, management support was a fundamental element in preserving mental capacity to work. The relevance of institutional support, promoted by nursing management, is highlighted in the construction of welcoming environments and attentive listening to difficulties, providing support to alleviate workers’ suffering and fears. The results of this work are consistent with a study carried out in Southeast Asia^(24)^ during the pandemic. The authors concluded that nurses with greater organizational and social support had fewer anxiety symptoms related to COVID-19.

These findings suggest that working directly with patients with COVID-19 represents significant psychological strain for nurses and adequate institutional support can prevent and alleviate psychological distress^(22)^ and be a protective factor for mental capacity to work.

Among the four variables in the health model, three were correlated with mental capacity to work. There was a direct relationship between physical and mental capacity to work. This finding is in line with a study carried out in Iran^(40)^, which showed a direct relationship between nurses’ physical effort and occupational cognitive failures. Factors external to the work environment, such as reduced physical activity before and during the pandemic, alter physical/emotional balance, with repercussions on the capacity to work^(16)^.

Through strong pressure, acute psychological stress activates the sympathetic system of the adrenal medulla and the hypothalamic-pituitary-adrenal axis, with consequences for physical and mental health^(41)^. National and international literature is vast regarding the mental and emotional disorders experienced by health professionals during the COVID-19 pandemic, with a negative emotional state associated with anxiety and anguish^(42)^, feelings of fear^(29)^, worsening of previous mental illness^(15)^, Burnout syndrome^(43)^, depressive symptoms and psychological distress^(13-14)^.

In the present study, the medically diagnosed illnesses self-reported by nursing technicians were anxiety, panic syndrome, mixed anxiety and depression disorder, anxiety disorder, depression and post-traumatic stress.

Pressure associated with prolonged stress culminated in the participating professionals’ mental illness, reducing their mental capacity to work. Similar results were identified in a study carried out in Turkey^(10)^, showing that health professionals’ problem-solving skills decreased with increasing anxiety levels. Other studies showed that nursing professionals experienced anxiety, depression and exhaustion during the pandemic, with impaired concentration, work capacity and professional performance^(24,43-44)^.

Research has shown that the nursing team caring for patients with COVID-19 had high levels of acute fatigue^(45)^, and inadequacy of resting places was associated with professional burnout^(46)^.

A review study^(47)^ showed that most professionals who worked in direct assistance to patients infected by coronavirus presented some type of skin impairment due to prolonged use of N95 mask or similar, protective glasses and face shield.

In the present study, model analysis chronology showed that including health-related variables (Model 3) increased explanatory power by 30.91%. It is likely that discontinued use of PPE due to skin injuries may have increased the fear of contamination and spread to family members^(12,35)^. Non-use of PPE due to skin injuries and lack of resting place may have contributed to the occurrence of emotional stress, which transformed, in the final model, into mental illness.

During the pandemic, mental and behavioral disorders may have been heightened. A study in Germany^(19)^ investigated the factors associated with sick leave of nurses in 2021, showing that the fear of becoming infected was associated with sick leave, being indicative of high mental vulnerability.

The present study revealed a negative correlation between mental capacity to work and the number of absences in 2021. A study carried out with nursing professionals before the pandemic already demonstrated that mental and behavioral disorders were the prevalent reasons for absences^(48)^.

The results of this study showed that hospital institutions, regardless of the pandemic, play a fundamental role in preserving nursing technicians’ health and mental capacity to work. Substantial improvements can be achieved with the qualification of nursing management to listen attentively and support workers, adaptation of places for rest and social interaction, implementation of workplace gymnastics to preserve physical capacity and adequate sizing of staff to minimize work overload.

### Study limitations

The hospitals were chosen for convenience and are of reference and excellence in health care, but they may not represent the work context of most Brazilian institutions. It is unknown whether the results of this study can be generalized to other health institutions.

Data collection was carried out in 2022, outside the height of the pandemic, and relied on participants’ memories. Due to the great impact of the health crisis on the lives of frontline workers, the authors understand that possible memory bias was not relevant.

The literature review revealed few studies during the pandemic involving factors associated with nursing technicians’ illness and work capacity. The comparison with the literature was carried out based on studies, mainly with nurses and physicians. The authors understand that this limitation may not provide generalized conclusions, but broaden the understanding of the topic.

### Contributions to nursing

The results contribute to understanding the work context during the pandemic, in particular to preserve the physical and mental capacity to work of nursing technicians.

As far as we know, this study was the first investigation, during the COVID-19 pandemic, into illness and the mental capacity to work of nursing technicians in Brazil. Thus, these findings are useful in providing initial information with the potential to support prevention and health protection actions.

## CONCLUSIONS

The study identified that physical capacity and management support were protective factors and contributed to preserving mental capacity to work.

Lack of tests for COVID-19, lack of knowledge of the routine and mental illness were factors that contributed to the worsening of mental capacity to work and increased absences in 2021.

Nursing professionals’ work directly compromises human life. Reducing their mental capacity to work in relation to mental demands can affect quality of care, with implications for patients and health institutions.

The authors understand that almost all factors identified in this study are under the governance of hospital management. The implementation of actions, often simple, can substantially improve the mental capacity to work of nursing technicians, reflected in quality of patient care.

Considering the above, it is suggested that new studies be carried out on the mental capacity to work of nursing technicians to complement and expand the understanding of the subject.
