# Supplementary material for: COVID-19 pandemic: what factors compromised the mental capacity to work of nursing technicians?
Source: Rev Bras Enferm. 2024 Jun 28;77(Suppl 1):e20220783. doi: 10.1590/0034-7167-2022-0783 (PMC11213538; doi:10.1590/0034-7167-2022-0783)
Supplement: Translation

# Translation: Pandemia COVID-19: que fatores comprometeram a capacidade mental para o trabalho dos técnicos de enfermagem?

**DOI:** 10.1590/0034-7167-2022-0783pt

**Authors:** Katia Maria Rosa Vieira, Francisco Ubaldo Vieira, Zélia Zilda Lourenço de Camargo Bittencourt

**Affiliations:** I Universidade Estadual de Campinas. Campinas, São Paulo, Brasil; II Instituto Federal de São Paulo. Campinas, São Paulo, Brasil

**Keywords:** Pandemia, COVID-19, Condições de Trabalho, Saúde do Trabalhador, Enfermagem

## Abstract

**Objetivos:**

identificar os fatores que influenciaram a capacidade mental para o trabalho de técnicos de enfermagem no contexto da pandemia da COVID-19.

**Métodos:**

estudo transversal, realizado em dois hospitais de referência no atendimento à população na pandemia da COVID-19. Participaram 237 profissionais de Unidades de Terapia Intensiva que responderam um questionário. Utilizaram-se modelos de regressão linear múltipla para avaliar a correlação entre a capacidade mental para o trabalho e variáveis relacionadas ao risco de contaminação, apoio institucional e saúde.

**Resultados:**

os fatores ausência de testes de COVID-19, desconhecimento da rotina, afastamentos em 2021 e adoecimento mental contribuíram para a piora da capacidade mental para o trabalho. O acolhimento da chefia e a capacidade física foram considerados fatores de proteção.

**Conclusões:**

a redução da capacidade para o trabalho em relação às exigências mentais pode afetar o desempenho profissional e a qualidade da assistência, com implicações para pacientes e instituições de saúde.

## INTRODUÇÃO

A saúde global foi ameaçada por uma doença denominada *Coronavirus Disease-2019* (COVID-19), desencadeada por um novo Coronavírus (SARS-CoV-2). Em virtude da sua expansão para todos os continentes do mundo, exceto a Antártida, a Organização Mundial da Saúde (OMS) declarou como pandemia em 11 de março de 2020^(1)^.

No decorrer da pandemia, órgãos internacionais e nacionais externaram sua preocupação com as condições de trabalho dos profissionais de saúde, prevenção de acidentes e promoção da cultura de segurança nas instituições^(2-3)^.

Os técnicos de enfermagem são a categoria que representa a maior força de trabalho em enfermagem no Brasil, com 1.715.191 profissionais (59,5%) com registros ativos^(4)^, e não tiveram recurso assistencial ou financeiro para lidar com o impacto do seu adoecimento ou de seus familiares durante essa grave crise sanitária^(4-5)^.

A pandemia da COVID-19 revelou fragilidades no sistema de saúde, que apresentou déficit de profissionais qualificados, ventiladores pulmonares, equipamentos de proteção individual (EPI), insumos essenciais^(6)^, ausência de testes para diagnóstico^(7)^ e falhas na organização das Unidades de Terapia Intensiva (UTI), com funcionamento superior à sua capacidade^(8)^.

Estudos realizados antes da pandemia demonstraram que o aumento da carga de trabalho intensificou o estresse e a ansiedade de médicos, enfermeiros e auxiliares de saúde, com diminuição das suas funções cognitivas, capacidade de raciocínio e julgamento, com prejuízo na atenção e coordenação^(9-10)^, com maiores chances de falhas e acidentes de trabalho^(11)^.

Pesquisas realizadas em vários países mostraram que, durante a pandemia, os profissionais de saúde apresentam sintomas de exaustão, ansiedade, estresse, depressão e adoecimento^(12-15)^, com redução da capacidade funcional dos enfermeiros com sintomas leves e infecções moderadas por COVID-19^(16-17)^ e comprometimento do desempenho profissional devido à excessiva carga de trabalho^(18)^, potencializada pelo absenteísmo^(19)^.

Esse panorama assumiu contorno complexo, pois, além de predispor os técnicos de enfermagem ao risco de contaminação pelo Coronavírus, comprometeu sua capacidade para o trabalho^(10,18,20)^. Esse conceito remete a um equilíbrio dinâmico entre estresse, condições do ambiente de trabalho, ferramentas e desgaste profissional^(21)^.

A capacidade mental para o trabalho dos técnicos de enfermagem está relacionada às suas funções cognitivas, que, para prestar assistência à saúde aos pacientes, requer capacidade de raciocínio e julgamento para solucionar problemas, memorizar informações, realizar cálculos e interpretar dados.

Estudo realizado nos Estados Unidos da América^(22)^ mostrou que os enfermeiros que trabalharam com pacientes com COVID-19 se sentiram desamparados, despreparados e sobrecarregados, com níveis elevados de esgotamento, sintomas físicos e emoções negativas.

A literatura internacional reporta algumas estratégias de apoio para prevenção e intervenção na saúde mental dos profissionais de enfermagem. Estudo realizado na China^(23)^ mostrou que estratégias cognitivas pautadas em valores e apoio social adequado facilitaram as experiências de trabalho com pacientes com COVID-19. Pesquisa realizada nas Filipinas, em 20 hospitais, revelou que o apoio organizacional e social teve efeitos consistentes na melhora da ansiedade dos enfermeiros^(24)^.

Na literatura atual, há poucos estudos específicos voltados aos técnicos de enfermagem no contexto da pandemia da COVID-19. É imperativo identificar os fatores associados à saúde mental e à capacidade para o trabalho da maior categoria profissional de atenção à saúde. Compreender essa relação permite ampliar o entendimento sobre as condições laborais, fornecendo subsídios para implementar ações de promoção à saúde e qualidade de vida desses trabalhadores.

## OBJETIVOS

Identificar os fatores que influenciaram a capacidade mental para o trabalho dos técnicos de enfermagem no contexto da pandemia da COVID-19.

## MÉTODOS

### Aspectos éticos

Foram seguidos todos os procedimentos éticos, conforme a Resolução n° 466/ 2012 do Conselho Nacional de Pesquisa. O projeto foi submetido e aprovado pelo Comitê de Ética em Pesquisa. Todos os participantes assinaram o Termo de Consentimento Livre e Esclarecido (TCLE), sendo respeitados o sigilo e a privacidade dos profissionais de enfermagem e instituições.

### Desenho, local de estudo e período

Trata-se de pesquisa de corte transversal, de caráter exploratório, de natureza quantitativa, norteado pelas diretrizes da ferramenta *STrengthening the Reporting of OBservational studies in Epidemiology* (STROBE).

O estudo foi desenvolvido em dois hospitais, selecionados por conveniência, que são centros de excelência na assistência médico-hospitalar e de referência no atendimento à população na pandemia da COVID-19, com as seguintes características: instituição 1 - hospital geral, público, estadual, de grande porte, de ensino e pesquisa, de nível terciário e quaternário, com atendimento exclusivo a usuários do Sistema Único de Saúde (SUS); instituição 2 - hospital geral, privado sem fins lucrativos, de grande porte, de ensino e pesquisa, de nível terciário, com atendimento a pacientes conveniados de planos de saúde e particulares.

As duas instituições de saúde se localizam na cidade de Campinas, interior do estado de São Paulo. Os dados foram coletados presencialmente pela pesquisadora no período de março a junho de 2022.

### População ou amostra: critérios de inclusão e exclusão

Todos os técnicos de enfermagem das instituições de saúde foram convidados a participar do estudo. No momento da coleta, os hospitais contavam com 384 técnicos de enfermagem em UTI, sendo que 56 encontravam-se em período de férias, licença ou afastamento. Somente oito sujeitos não aceitaram participar: seis do hospital público e dois da rede privada.

Considerou-se como critérios de inclusão todos os técnicos de enfermagem que atuaram por, no mínimo, seis meses nas UTI das instituições, de ambos os sexos, que prestaram assistência direta aos pacientes com COVID-19 nos turnos de trabalho matutino, vespertino, noturno. Foram excluídos os profissionais que estavam em período de férias, licença ou afastamento.

### Protocolo de estudo

Para a coleta de dados, desenvolveu-se um questionário, do tipo *checklist*, com perguntas objetivas. Aplicou-se um pré-teste em seis sujeitos de uma das instituições participantes, sendo o instrumento posteriormente readequado nos aspectos semântico e sintáxico.

O questionário foi constituído por quatro blocos estruturados de perguntas.

Bloco 1 - Dados gerais e sociodemográficos: sexo; idade; tempo de instituição; tempo de trabalho em UTI; e número de empregos.

Bloco 2 - Fatores de risco de contaminação pela COVID-19, podendo o participante elencar um ou mais itens: jornada de trabalho prolongada; ritmo de trabalho acelerado; falta de EPI; má qualidade dos EPI; excesso de empregos (dois ou mais); ausência e/ou demora na vacinação dos profissionais da saúde; falta de local adequado para descanso; não uso de EPI; acidentes de trabalho com fluído biológico/secreção respiratória de paciente com COVID-19; falta de informações seguras; déficit/ausência de testes para trabalhador com suspeita de COVID-19; sobrecarga de trabalho; número insuficiente do quadro de pessoal de técnicos de enfermagem; estresse; faltas nos plantões (absenteísmo); falta e/ou falha na higienização das mãos; desconhecimento da rotina de trabalho da UTI; falha na circulação entre área contaminada e não contaminada; saída/deslocamento acidental de EPI (queda da máscara, outros); falta de treinamento/educação em serviço; falha na paramentação/desparamentação do avental; uso descontínuo de EPI devido ao aparecimento de lesões de pele na pandemia; falha no descarte de resíduos hospitalares; falta e/ou falha na limpeza e desinfecção de superfícies; contaminação durante manipulação do EPI (máscaras N95/PFF2).

Bloco 3 - Medidas institucionais de apoio aos profissionais de saúde, podendo o participante elencar um ou mais itens: apoio de psicólogo; apoio de psiquiatra; acolhimento da chefia; apoio de equipe multiprofissional; ginástica laboral; e práticas integrativas.

Bloco 4 - Saúde do trabalhador: doenças adquiridas na pandemia com diagnóstico médico (padrão de resposta: Sim-Não, com descrição do diagnóstico); afastamentos do trabalho durante os anos de 2020 e 2021 (padrão de resposta: Sim-Não); autopercepção da capacidade mental e física para o trabalho atual em relação às exigências mentais (solucionar problemas, realizar cálculos, memorizar informações, analisar dados) e exigências físicas (realizar esforço físico, manipular pacientes, realizar transportes), em comparação com antes da pandemia da COVID-19 (padrão de resposta: Piorou muito = 1, Piorou = 2, Mesma capacidade = 3, Melhorou = 4 e Melhorou muito = 5).

### Análise dos resultados e estatística

Após a coleta, os dados foram digitalizados na planilha do programa *Microsoft Excel* ^®^ (v.2308), e a análise estatística foi realizada com o auxílio do programa Minitab 19. Utilizou-se a regressão linear múltipla, com procedimento gradual e seleção automática *stepwise* para examinar a correlação entre variável dependente capacidade mental para o trabalho e conjunto de variáveis independentes, incluídas nos seguintes modelos:

Modelo 1 - (Risco de contaminação): devido ao grande número de variáveis independentes (25) relacionadas aos fatores de risco de contaminação (bloco 2), utilizou-se o coeficiente Cp de Mallows para auxiliar no equilíbrio do número de preditoras (variáveis) que melhor se ajustassem à capacidade mental para o trabalho. Um valor de Cp de Mallows que está próximo do número de preditores mais a constante indica que o modelo é relativamente imparcial na estimativa dos coeficientes de regressão verdadeiros e não viciados. Para isso, utilizou-se Cp ≈ K+1, sendo que k - foi o número de variáveis do modelo.

Modelo 2 - (Apoio institucional): foram incluídas as variáveis apoio de psiquiatra, apoio psicológico, acolhimento da chefia, apoio da equipe multiprofissional, ginástica laboral e práticas integrativas para medir a influência do apoio institucional na capacidade mental para o trabalho.

Modelo 3 - (Saúde): foram incluídas as variáveis capacidade física para o trabalho, afastamentos em 2020, afastamentos em 2021 e adoecimento mental.

A colinearidade foi avaliada pelo fator de inflação de variância (FIV), sendo que FIV<5 foi interpretado como ausência de colinearidade. Utilizou-se o teste de Shapiro-Wilk, para verificar a normalidade dos resíduos, o teste de falta de ajuste, para avaliar a adequação dos dados ao modelo, o teste de Durbin-Watson, para verificar a autocorrelação dos resíduos, e o coeficiente de determinação ajustado (R^2^_aj_), para avaliar a qualidade de ajuste dos modelos. Para todos os resultados, considerou-se p < 0,05 como estatisticamente significante.

## RESULTADOS

Participaram da pesquisa 237 técnicos de enfermagem (179 mulheres, 58 homens), sendo 143 de instituição pública e 94 de instituição privada, com média de idade de 38,4±9,8 anos (média ± desvio padrão), tempo de instituição de 7,8±6,6 anos, tempo de trabalho em UTI de 6,0±5,4 anos e média do número de empregos de 1,5±0,55. Dos participantes, 64,3% tiveram autopercepção de piora da capacidade mental atual para o trabalho, e 67,2 %, de piora da capacidade física atual para o trabalho, em comparação com antes da pandemia da COVID-19.

A análise dos melhores subconjuntos para as variáveis incorporadas no Modelo 1, relacionadas ao risco de contaminação, incluiu estresse, falta de local de descanso, ausência de testes para COVID-19, desconhecimento da rotina, contaminação na manipulação de EPI e não uso de EPI devido a lesões de pele. Esse foi o melhor subconjunto com seis variáveis, e resultou no Cp de Mallows = 6,98.

A Tabela 1 mostra os resultados da regressão linear múltipla das variáveis que compuseram os três modelos.

**Tabela 1:** Regressão linear múltipla da capacidade mental para o trabalho de técnicos de enfermagem em relação às variáveis contidas nos modelos, Campinas, São Paulo, Brasil, 2023

Variável	Capacidade mental para o trabalho					
	Modelo 1 Risco de contaminação		Modelo 2 Apoio institucional		Modelo 3 Saúde	
	Coef	FIV	Coef	FIV	Coef	FIV
Estresse	-0,245^^*^^	1,10	-0,234^^*^^	1,10	--	--
Falta de local de descanso	-0,206	1,10	-0,205^^*^^	1,13	--	--
Ausência de testes para COVID-19	-0,294^^*^^	1,10	-0,243^^*^^	1,14	-0,277^†^	1,11
Desconhecimento da rotina	-0,251	1,10	-0,245	1,10	-0,212^^*^^	1,06
Contaminação na manipulação de EPI	-0,211	1,10	-0,213	1,13	--	--
Não uso de EPI devido a lesões de pele	-0,371^^*^^	1,10	-0,393^^*^^	1,13	-0,258	1,10
Acolhimento da chefia			0,277^^*^^	1,10	0,192^^*^^	1,07
Apoio da equipe multiprofissional			0,310^^*^^	1,03	0,206	1,07
Capacidade física para o trabalho					0,439^†^	1,12
Afastamentos em 2020					-0,170	1,23
Afastamentos em 2021					-0,263^†^	1,17
Adoecimento mental					-0,366^†^	1,07
R^2^	12,48%		16,42%		47,33%	
R^2^_aj_	10,20%		13,49%		46,58%	
Normalidade (Shapiro-Wilk)	<0,01		<0,01		>0,100	
Falta de ajuste	0,930		0,378		0,686	
Durbin-Watson	> 0,05		> 0,05		> 0,05	

* *p<0,05; †p<0,01; Coef - coeficiente de inclinação; FIV - fator de inflação de variância; EPI - equipamentos de proteção individual.*

Os resultados da regressão linear múltipla das variáveis que compuseram o risco de contaminação (Modelo 1) apresentaram baixo poder explicativo (R^2^_aj_=10,2%), revelando que as variáveis estresse, ausência de testes para COVID-19 e não uso de EPI devido a lesões de pele foram estatisticamente significantes e mostraram correlação negativa com a capacidade mental para o trabalho.

Com a inclusão das variáveis que compuseram o apoio institucional (Modelo 2), melhorou o poder explicativo da capacidade mental para o trabalho (R^2^_aj_=13,49%), revelando que as variáveis acolhimento da chefia e apoio da equipe multiprofissional apresentaram significância estatística. As variáveis do risco de contaminação, estresse, ausência de testes para COVID-19 e não uso de EPI devido a lesões de pele mantiveram significância, e a variável falta de local de descanso passou a compor o modelo. Ressalta-se que as variáveis relacionadas ao apoio institucional mostraram correlação positiva com a capacidade mental para o trabalho.

Ao se adicionar as variáveis relacionadas à saúde (Modelo 3), houve melhora substancial na capacidade explicativa da capacidade mental para o trabalho (R^2^_aj_ = 46,58%), revelando que as variáveis capacidade física para o trabalho, afastamentos em 2021 e adoecimento mental foram estatisticamente significantes. Ressalta-se que, com a inclusão das variáveis relacionadas à saúde, as variáveis estresse, falta de local de descanso e contaminação na manipulação de EPI passaram a não compor mais o modelo, e as variáveis não uso de EPI devido a lesões de pele e apoio da equipe multiprofissional não mostraram mais significância estatística. Com a inclusão das variáveis relacionadas à saúde, o desconhecimento da rotina passou a compor o modelo final.

No modelo final, não houve indicação de que a multicolinearidade viciou os resultados, pois nenhuma das pontuações do FIV excedeu 1,3. O teste de normalidade dos resíduos mostrou distribuição normal (p>0,10), e não houve indicação de autocorrelação dos resíduos (p>0,05) ou falta de ajuste dos dados (p>0,686).

O modelo final ficou representado pelas seguintes variáveis: ausência de testes para COVID-19; desconhecimento da rotina; acolhimento da chefia; capacidade física para o trabalho; afastamentos em 2021; e adoecimento mental.

Observou-se, no modelo final, que as variáveis incorporadas ao risco de contaminação tiveram seus coeficientes de correlação negativos, isto é, comprometeram negativamente a capacidade mental para o trabalho. A variável que compôs o apoio institucional (acolhimento de chefia) mostrou correlação positiva com a capacidade mental. Por último, as variáveis que compuseram o modelo saúde mostraram correlação positiva entre a capacidade física e a capacidade mental. Já o afastamento 2021 e o adoecimento mental se correlacionaram negativamente com a capacidade mental para o trabalho.

## DISCUSSÃO

A crise pandêmica impactou profundamente o mundo, devido à rápida disseminação da doença e aos desafios impostos às instituições de saúde. Houve um descompasso na velocidade de construção de evidências científicas e implementação de ações efetivas ao longo do tempo que, muitas vezes, traduziram-se no aumento das chances de contaminação da equipe de enfermagem, com impactos na saúde física e mental do trabalhador, bem como na qualidade da assistência^(25)^.

No Brasil, um dos países mais afetados pela pandemia, a situação foi agravada em virtude de divergências, desalinhamento de ações governamentais e visões distintas nas formas de enfrentamento da crise sanitária. Na gestão pública, adotou-se um protocolo para uso de cloroquina e hidroxicloroquina para tratamento dos pacientes com COVID-19, protagonizado pelo Governo Federal brasileiro. Em virtude da falta de lastro científico para justificar a adoção desse tratamento, ocorreram embates e contradições entre profissionais de saúde, sociedades científicas e instituições públicas de autoridade sanitária^(26)^.

Apesar das divergências, a COVID-19 se disseminou no país, com elevadas taxas de morbimortalidade^(27)^. Os profissionais de saúde que estavam na linha de frente se empenharam na assistência aos pacientes, com poucos recursos e conhecimento^(6)^, alto risco de exposição ocupacional^(2)^, além da redução da capacidade de atenção^(9)^ e de solução de problemas no trabalho^(10)^.

O presente estudo analisou 25 variáveis relacionadas ao risco de contaminação. Apenas a ausência de testes para COVID-19 e o desconhecimento da rotina tiveram correlação com a capacidade mental para o trabalho.

Para enfrentar uma crise sanitária dessa magnitude, foram necessários profissionais de enfermagem com aptidões físicas e mentais para realizar as atividades em um ambiente permeado por condições laborais inadequadas^(15)^. Pesquisas mostraram que a jornada prolongada^(18)^ e o ritmo de trabalho acelerado^(20)^ foram associados à redução da capacidade para o trabalho. Entretanto, no presente estudo, esses resultados não foram observados.

Os profissionais de saúde vivenciaram forte estresse emocional^(12)^, pressões psicológicas e incertezas das proporções da pandemia^(28)^, principalmente devido à falta de testes diagnósticos para COVID-19^(29)^, medo do risco de contaminação e disseminação da doença para familiares, falta de informação e possibilidade de transtornos mentais^(30-31)^.

Na presente pesquisa, a ausência de testes para COVID-19 se relacionou, negativamente, à capacidade mental para o trabalho, incrementando a insegurança e o receio de disseminação do Coronavírus, e, quando associada ao estresse, contribuiu para redução das funções cognitivas para o trabalho.

Esse resultado é consistente com a literatura. Estudo desenvolvido na Polonia^(32)^ mostrou que a possibilidade de realização de testes para COVID-19 influenciou de forma protetora a saúde mental dos enfermeiros. Pesquisa na Indonésia^(7)^, com enfermeiros, mostrou associação entre o estresse e a falta de serviços de testes diagnóstico para COVID.

A incerteza e a escassez de conhecimento inicial sobre a COVID-19 causaram forte estresse e sentimentos de pânico entre a população em geral^(33)^. O desconhecimento sobre o período de incubação do vírus, sua forma de transmissão, tratamento e medidas de segurança causou sentimentos de medo e ansiedade, com impactos negativos para a saúde mental devido à incerteza com relação ao futuro^(34)^.

O trabalho da enfermagem é caracterizado por rotinas e protocolos bem estabelecidos nos serviços de saúde. Durante a pandemia, houve alteração substancial dessas rotinas, com extensão das jornadas laborais^(35)^, falta de informação sobre a nova doença^(29)^, incapacidade do sistema de saúde de atender à demanda^(8)^, treinamentos frequentes^(13)^, novos protocolos de biossegurança^(12)^ e falta de conhecimento para cuidar dos pacientes^(36)^.

No presente estudo, o desconhecimento da rotina de trabalho foi uma variável relacionada negativamente à capacidade mental para o trabalho. No contexto da pandemia, o país vivenciava uma infodemia^(37)^ relacionada aos protocolos de prevenção e controle para o combate ao vírus que pode ter potencializado o adoecimento mental dos técnicos de enfermagem.

Esse achado foi consistente com estudo realizado na China^(38)^, no qual os enfermeiros que estavam na linha de frente do combate ao Coronavírus apresentaram medo, ansiedade, raiva e tristeza devido ao contato próximo com os pacientes, à falta de familiaridade com procedimentos e locais de trabalho incomuns. Essas condições desencadearam sofrimento psíquico, causando sintomas depressivos, de ansiedade e de estresse^(14)^ que afetaram o desempenho e a capacidade mental para o trabalho.

No presente trabalho, entre as variáveis que compuseram o modelo relacionado ao apoio institucional, apenas o acolhimento da chefia apresentou correlação positiva com a capacidade mental para o trabalho.

O bem-estar dos integrantes de equipes multidisciplinares é uma responsabilidade compartilhada entre a instituição de saúde e os profissionais que a integram. No presente estudo, os integrantes das equipes multiprofissionais estavam expostos a inúmeros estressores no ambiente laboral^(12,15)^, com divergências de condutas e dificuldades na comunicação. Isso impossibilitou que o apoio da equipe multiprofissional se tornasse um fator de proteção para a capacidade mental para o trabalho.

Esse resultado é consistente com estudo realizado na Austrália^(39)^ durante a pandemia da COVID-19 com equipe multiprofissional composta por médicos, enfermeiros e profissionais de apoio. As descobertas destacaram que os profissionais de enfermagem apresentaram menor satisfação com o ambiente de laboral, e a falta de apoio da equipe contribuiu para maiores níveis de estresse, ansiedade e esgotamento.

O apoio organizacional foi identificado na literatura como fator de proteção para as adversidades e o estresse dos enfermeiros, permitindo-lhes manter o seu bem-estar e saúde mental^(24)^.

Na presente pesquisa, o acolhimento de chefia foi um elemento fundamental para preservar a capacidade mental para o trabalho. Destaca-se a relevância do apoio institucional, promovido pelas gerencias de enfermagem, na construção de ambientes acolhedores e escuta atenciosa das dificuldades, proporcionando suporte para alívio do sofrimento e temores dos trabalhadores. Os resultados do presente trabalho são consistentes com estudo realizado no sudeste asiático^(24)^ durante a pandemia. Os autores concluíram que os enfermeiros com maior apoio organizacional e social apresentavam menos sintomas de ansiedade relacionados à COVID-19.

Essas descobertas sugerem que trabalhar diretamente com pacientes com COVID-19 representa tensão psicológica significativa para os enfermeiros e o apoio institucional adequado pode prevenir e amenizar o sofrimento mental^(22)^ e ser um fator de proteção da capacidade mental para o trabalho.

Entre as quatro variáveis do modelo de saúde, três tiveram correlação com a capacidade mental para o trabalho. Houve relação direta entre capacidade física e mental para o trabalho. Esse achado está em conformidade com estudo realizado no Irã^(40)^, que mostrou relação direta entre o esforço físico do enfermeiro e as falhas cognitivas ocupacionais. Os fatores externos ao ambiente laboral, como redução da prática de atividades físicas antes e durante a pandemia, alteram o equilíbrio físico/emocional, com repercussão sobre a capacidade para o trabalho^(16)^.

Mediante forte pressão, o estresse psicológico agudo ativa o sistema simpático da medula adrenal e o eixo hipotálamo-hipófise-adrenal, com consequências sobre a saúde física e mental^(41)^. A literatura nacional e internacional é vasta quanto aos transtornos mentais e emocionais vivenciados pelos profissionais de saúde na pandemia da COVID-19, com estado emocional negativo associado a ansiedade e angústia^(42)^, sentimento de medo^(29)^, piora de quadros prévios de adoecimento mental^(15)^, síndrome de *Burnout* ^(43)^, sintomas depressivos e sofrimento psíquico^(13-14)^.

No presente estudo, as doenças com diagnóstico médico autorreferidas pelos técnicos de enfermagem foram ansiedade, síndrome do pânico, transtorno misto de ansiedade e depressão, transtorno de ansiedade, depressão e estresse pós-traumático.

A pressão associada ao estresse prolongado culminou no adoecimento mental dos profissionais participantes, e reduziu sua capacidade mental para o trabalho. Resultados semelhantes foram identificados em estudo realizado na Turquia^(10)^, evidenciando que as habilidades de resolução de problemas dos profissionais de saúde diminuíram com o aumento dos níveis de ansiedade. Outros estudos mostraram que os profissionais de enfermagem apresentaram ansiedade, depressão e esgotamento durante a pandemia, com prejuízo na concentração, na capacidade de trabalho e no desempenho profissional^(24,43-44)^.

Pesquisas mostraram que a equipe de enfermagem que cuidava de pacientes com COVID-19 teve níveis elevados de fadiga aguda^(45)^, e a inadequação dos locais de descanso foi associada ao esgotamento dos profissionais^(46)^.

Estudo de revisão^(47)^ evidenciou que a maior parte dos profissionais que atuaram na assistência direta aos pacientes infectados por Coronavírus apresentou algum tipo de comprometimento na pele devido ao uso prolongado da máscara N95 ou similar, óculos de proteção e *face shield*.

No presente estudo, a cronologia de análise dos modelos mostrou que a inclusão das variáveis relacionadas à saúde (Modelo 3) incrementou 30,91% no poder explicativo. É provável que o uso descontinuado de EPI devido a lesões de pele pode ter incrementado o medo de contaminação e disseminação para os familiares^(12,35)^. Os fatores não uso de EPI devido a lesões de pele e falta de local de descanso podem ter contribuído para o incremento do estresse emocional, que se transformou, no modelo final, em adoecimento mental.

Durante a pandemia, os transtornos mentais e comportamentais podem ter sido potencializados. Estudo na Alemanha^(19)^ investigou os fatores associados aos afastamentos por licença médica de enfermeiros em 2021, mostrando que o medo de se infectar foi associado aos afastamentos, sendo indicativo de vulnerabilidade mental elevada.

O presente estudo revelou correlação negativa entre a capacidade mental para o trabalho e o número de afastamentos em 2021. Estudo realizado com profissionais de enfermagem antes da pandemia já demonstrava que transtornos mentais e comportamentais eram os motivos prevalentes de afastamentos^(48)^.

Os resultados deste estudo mostraram que as instituições hospitalares, independentemente da pandemia, têm papel fundamental para preservar a saúde e a capacidade mental para o trabalho dos técnicos de enfermagem. Melhoras substanciais podem ser alcançadas com a qualificação das gerencias de enfermagem para escuta atenciosa e apoio aos trabalhadores, adequação de locais de descanso e convívio social, implementação de ginástica laboral para preservar a capacidade física e dimensionamento adequado do quadro de pessoal para minimizar a sobrecarga laboral.

### Limitações do estudo

Os hospitais foram escolhidos por conveniência, e são de referência e excelência na assistência à saúde, mas eles podem não representar o contexto de trabalho da maioria das instituições brasileiras. Não se sabe se os resultados deste estudo podem ser generalizados para outras instituições de saúde.

A coleta de dados foi realizada em 2022, fora do auge da pandemia, e contou com a memória dos participantes. Devido ao grande impacto da crise sanitária na vida dos trabalhadores da linha de frente, os autores entendem que o possível viés de memória não foi relevante.

A revisão bibliográfica revelou poucos estudos durante a pandemia envolvendo fatores associados ao adoecimento e à capacidade para o trabalho de técnicos de enfermagem. O cotejamento com a literatura foi realizado a partir de estudos, principalmente, com enfermeiros e médicos. Os autores entendem que essa limitação pode não fornecer conclusões generalizadas, mas ampliar a compreensão sobre a temática.

### Contribuições para a área da enfermagem

Os resultados contribuem para o entendimento do contexto laboral durante a pandemia, em especial, para preservar a capacidade física e mental para o trabalho de técnicos de enfermagem.

Até onde sabemos, este estudo foi a primeira investigação, durante a pandemia da COVID-19, sobre o adoecimento e a capacidade mental para o trabalho de técnicos de enfermagem no Brasil. Assim, essas descobertas são úteis para fornecer informações iniciais com potencial para subsidiar ações de prevenção e proteção à saúde.

## CONCLUSÕES

O estudo identificou que a capacidade física e o acolhimento da chefia foram fatores de proteção e contribuíram para preservar a capacidade mental para o trabalho.

A ausência de testes para COVID-19, o desconhecimento da rotina e o adoecimento mental foram fatores que contribuíram para a piora da capacidade mental para o trabalho e potencializaram os afastamentos em 2021.

O desempenho dos profissionais de enfermagem compromete diretamente a vida humana. A redução da sua capacidade mental para o trabalho em relação às exigências mentais pode afetar a qualidade da assistência, com implicações para os pacientes e instituições de saúde.

Os autores entendem que quase todos os fatores identificados nesse estudo estão sob a governança da gestão hospitalar. A implementação de ações, muitas vezes simples, pode melhorar substancialmente a capacidade mental para o trabalho dos técnicos de enfermagem, com reflexo na qualidade da assistência aos pacientes.

Diante do exposto, sugere-se a realização de novos estudos sobre a capacidade mental para o trabalho de técnicos de enfermagem para complementar e ampliar o entendimento sobre a temática.
